# Method to plan, administer, and verify supine craniospinal irradiation

**DOI:** 10.1120/jacmp.v3i4.2555

**Published:** 2002-09-01

**Authors:** Jeff M. Michalski, Eric E. Klein, Russell Gerber

**Affiliations:** ^1^ Department of Radiation Oncology, Mallinckrodt Institute of Radiology Washington University School of Medicine St. Louis Missouri 63110; ^2^ Present address: Department of Radiation Oncology Mallinckrodt Institute of Radiology, Washington University Medical School 4939 Children's Place, Suite 5500 St. Louis MO 63110

**Keywords:** craniospinal, radiotherapy, treatment verification

## Abstract

Craniospinal irradiation remains an important technique in the management of malignancies of the central nervous system. It is technically demanding, with potential for treatment field overlap or gaps to yield unacceptable dosimetric heterogeneity. A method to accurately simulate and verify the three‐field junction is described. We use a comfortable supine position to minimize patient movement. The supine position provides airway access by anesthesiology in patients requiring sedation or anesthesia. Virtual simulation is performed with a dedicated computed tomography (CT) simulator. Multiplanar sagittal and coronal CT reconstructions allow visual confirmation of three‐field matching at the cervical region. The placement of isocenters for each field, table position, and collimator angles are determined by calculation of field sizes accommodating for beam divergence. At treatment, exact matching of the three fields is assured using the record and verify confirmation of beam collimator settings and rotation, digital couch readouts, and gantry parameters. Mini‐verification silver halide (Kodak XV) films (6×6cm) are placed behind the patient's neck and are exposed by all treatment fields (posterior flash from the lateral cranial fields and entrance from the PA spine field). These films assess field placement accuracy at the junction of these three fields. Finally, placement of radio‐opaque markers at the junction is visualized in each clinical portal radiograph. Patients readily accept the supine position as their treatment setup is eased. Field placement using digital couch settings is efficient and accurate. Daily mini‐verification films are simple, inexpensive, and allow verification of each treatment field matching. Field placement errors of greater than 1 mm can be readily identified and corrected at subsequent treatment sessions. Virtual simulation and direct junction verification with mini‐verification films allow for simple and quantitative evaluation of the junction associated with the three‐field craniospinal irradiation technique. The supine patient position does not present any difficulties in field matching or verification.

PACS number(s): 87.53.–j, 87.53.–j

## BACKGROUND AND INTRODUCTION

Craniospinal irradiation (CSI) remains an important element in the management of several malignant neoplasms that affect the central nervous system. Medulloblastoma and other intracranial primitive neuroectodermal tumors, ependymomas, gliomas, or leukemias may seed the neuroaxis and are effectively or prophylactically treated with CSI.

Many methods have been described to deliver craniospinal irradiation.^1^ ^–^ ^5^ Often technical constraints have limited the flexibility with which this treatment was administered. Treatment couch range limited the length for posterior spine field(s), and patients were therefore treated in prone position. One advantage of the prone position is that it allows direct visual confirmation of the junction between the lateral brain fields and posterior spine field. On occasion a single spine field will suffice for pediatric cases.

Unfortunately, the prone position has some significant disadvantages. Many patients are uncomfortable, and their tolerance for the long simulation process is limited. Access to the oral cavity and airways is restricted, a definite problem for young children requiring sedation or anesthesia. In addition, the flexed head position is not conducive to immobilization.

Recent modifications of treatment tables to accommodate long field sizes and asymmetric or independent collimation, virtual simulation technology and record and verify computers, have simplified the simulation, treatment delivery, and verification process for this complex treatment technique. This report details our technique that uses these technological advances.

## METHODS

Our technique involves virtual simulation, followed by verification simulation, followed by treatment. The field arrangement is a variation on the method described by van Dyk *et al.* ^1^ Field matching is accomplished by use of independent, asymmetric jaws, collimator rotation, and table angulation.^2^ After five fraction intervals, the field junctions are shifted 1 cm by changing the independent collimator jaw settings.

### Simulation

Initial simulation begins with patient positioning and immobilization. Patients are placed supine with their heads on a modified headrest that extends their necks enough to minimize exit of the superior edge of the superior spine field into the oral cavity. An immobilization mask is fabricated from thermal plastic material. In patients requiring anesthesia, an airway opening is cut out of the mask over the mouth. The immobilization masks are fastened to a thin Lucite platform that fixates to the tables of the CT scanner, verification simulator, or treatment table. This allows co‐ordinate assignment and registration of the patient throughout simulation and treatment process.

CT scans range from the top of the head to the mid‐pelvis. CT spacing and thickness vary from 3mm×3mm to 5mm×5mm, respectively. The 3 mm slices are done through the cervical and upper lumbar spine where high quality digital reconstruction radiographs (DRRs) are necessary to verify cervical junctions and lumbar spine field gaps. Two hundred or more CT slices are typically acquired in this simulation procedure. Patients are marked with sagittal and lateral laser lines to facilitate repositioning during the verification simulation and subsequent treatment. Positioning laser marks are placed at the $C_{2}–C_{3}$ interspace where the first junction between cranial and spinal fields is anticipated. Another set of positioning laser marks is placed mid‐trunk, corresponding to the position of the anticipated isocenter of the spine field. This laser marking may change at the verification simulation once the final isocenter is chosen. The CT acquisition generally takes 30 to 45 min. This is a sufficiently short time that minimizes patient discomfort and anesthesia risk.

The digital CT scans are then transferred to a VoxelQ virtual simulation workstation (Picker Medical Systems, Cleveland, OH). In a method analogous to conventional simulation, treatment fields are designed for treatment. First, the posterior spine fields are simulated, and the superior beam divergence is calculated. The superior border of the spine field is initially placed at the $C_{2-3}$ interspace (or lower to avoid exit radiation through the oral cavity). If necessary, an inferior spine field is simulated with an appropriate gap on the posterior skin surface in order to match the fields at the depth of the spine. The gap and divergence can be visualized by a sagittal reconstruction. Cranial ports are then planned with collimator rotations to match the superior spine field's divergence. The isocenter of the cranial fields is established at midplane just behind the eyes. This placement minimizes beam divergence into the contralateral lens.^3^ Because the inferior divergence of the cranial fields may overlap into the spinal cord, a treatment table rotation is calculated to effectively have all three field edges from both lateral brain ports and the posterior spine port to meet at the cervical junction as a single plane.

The formulas to calculate these divergences are

(1) $$\Theta_{Cr,Col}=\tan^{-1}\frac{Y_{2,US}}{SAD}$$

for the appropriate cranial collimator rotation and

(2) $$\Theta_{Cr,table}=\tan^{-1}\frac{Y_{1,Cr}}{SAD}$$

for the appropriate cranial table rotation, where $Y_{2,\text{US}}$= superior collimator setting of upper spine field, and $Y_{1,\text{Cr}}$ = inferior collimator setting of cranial field.

The field edge matching can be visually verified as the junctions share a common, overlapping “plane.” Unlike older methods that used symmetric collimator jaw settings, the total length of the fields should not be used when calculating the appropriate diverging angles. On the virtual simulator workstation, the distance between the two isocenters (three if two spine fields are required) can be calculated once the beams have been set. This distance can then be used as the digital longitudinal table distance shift. Custom shields protecting normal tissue are then created using the virtual simulator field shaping software. It is our preference to use cerrobend shielding in this site due to the fine detail required to shield sensitive structure in close proximity to the target volume. When 5 mm multileaf collimators become available in our clinic, we may use them in this situation. Design of the fields is facilitated with anatomic definition of eyes and the brain at the cribriform plate and middle cranial fossa.

**Figure 1:**
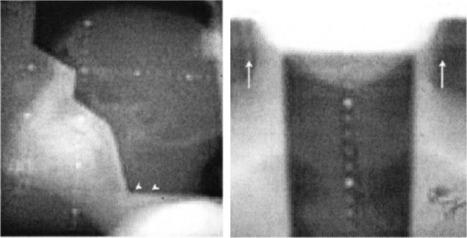
Clinical portal radiographs of a lateral cranial field and posterior (upper) spine field. Lead beads can be seen at the inferior field edge of the cranial portals and at the top level of the upper spine portal on the double exposure. The lead beads are taped to the immobilization mask at the level of $C_{2}$.

Patients return to a conventional simulator after block fabrication for a “verification simulation.” Isocenters determined at the virtual simulation process are verified and marked. Registered coordinates for digital table positions are assigned during the virtual simulation process. Shifts from the positioning laser marks to the treatment isocenters are described on a “set‐up” sheet for the therapists. Films are taken at the verification simulation and compared to the DRR from the virtual simulation. The verification simulation takes approximately 1 h, 30 to 60 min less time than previously dedicated to the same process after a conventional simulation. The table and immobilization devices are inspected to assure they do not obstruct or shadow the spine field. Lead beads are placed on the immobilization mask at the level of the cervical three‐field junction. These markers are visible on clinical portal radiographs to assist in verifying the junction placement during therapy (Fig. 1).

The gantry angle, collimator angle, table angle, asymmetric jaw settings, and table positions (vertical, lateral, and longitudinal) are digitally acquired during the verification simulation into the record and verify system (Varis, Varian Oncology, Palo Alto, CA) for treatment. The future treatment parameters accounting for scheduled junction shifts are also programmed into the record and verify system and scheduled appropriately. The record and verify system provides a large in‐room screen display of the nominal values and allows auto‐setup functions of the jaw and collimator settings. Setup deviations greater than 1 mm or 1 ° require user password intervention.

### Treatment

The longitudinal distance between each isocenter can be calculated using straightforward geometric and trigonometric principles. These calculations may be verified using the “ruler tool” on the virtual simulation software or by the difference between the two isocenters' (three if two spine fields) couch vertical coordinates. They are also verified at the verification simulation. Isocenter marks on the mask corresponding to the cranial fields are placed after review and approval of films taken at the verification simulation. Setup marks are also placed on the patient's trunk corresponding to the spinal fields in the same manner. Filming at the verification simulation provides hard copy film documentation of both adequate block design and field localization. Therefore, “preporting” at the linear accelerator is not required unless changes in block shaping or field localization are required. After the first day's treatment, portal radiographs are evaluated. The position of the lead beads at the cervical junction is verified in each of the lateral cranial and posterior superior spine radiographs. Coordinates of both the cranial and spinal field isocenters are recorded into Varis once satisfactory localization is determined by review of portal and match films. Table position tolerances are then restricted to 1 mm in order to maintain appropriate field junctions and gaps at the cervical and lumbar spine regions, respectively.

Visual verification of the junction is possible. The inferior border of the cranial fields can be marked on the mask or the patient's neck. When the PA spine field has been set up, the x‐collimator (lateral) can be opened wide to allow light field to illuminate on the neck through the Mylar sheet or “tennis racket” of the treatment table. Once the superior border of the spine field matching the inferior border of the cranial field is confirmed, the lateral borders are re‐set to the actual width of the spine field before treatment.

## MINI‐VERIFICATION FILMS

**Figure 2:**
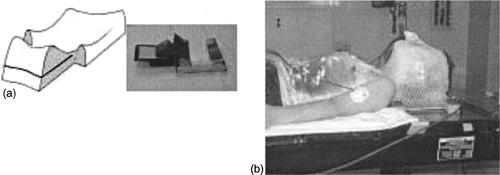
(a) A modified headrest allows placement of a mini‐verification film under the patient's neck. (b) Patient in treatment position with his head on the headrest. The mini‐verification film rests posterior to his neck. Lead beads are taped to the mask at $C_{2}$.

To further confirm proper field matching, the headrest for CSI has been modified to hold a $6\times 6{\text{cm}}^{2}$ piece of Kodak XOMAT V film. These mini‐films are prepared in a darkroom and sealed with opaque tape. At each daily treatment, a mini‐film is placed in a horizontally sliced opening in the headrest. This film lies directly behind the patient's neck at the $C_{2-5}$ vertebral body levels (Fig. 2).

The film is exposed during treatment by the posterior flash of the inferior border of each cranial field and the entrance of the superior field edge of the upper spine field. The mini‐films are developed and examined daily to verify the three‐field junction. The processing of these mini‐films requires taping them to a large standard size film to prevent jamming within the film processor. The films are reviewed by a clinic physicist or the treating radiation oncologist prior to the next day's treatment. All overlaps are corrected. Gaps in the junction of 3 mm or more are corrected. Gaps of 2 mm or less are considered ideal.

To illustrate the utility of the mini‐verification films, we irradiated a solid water phantom with the verification films placed in craniospinal treatment geometry. Both ideal and incorrect setups were used. These films were then scanned with a film densitometer to demonstrate the magnitude of dose error with incorrect setups. The densities were converted to dose by corrections according to dose/density film conversion film sets acquired at the time of measurement.

## RESULTS

**Figure 3:**
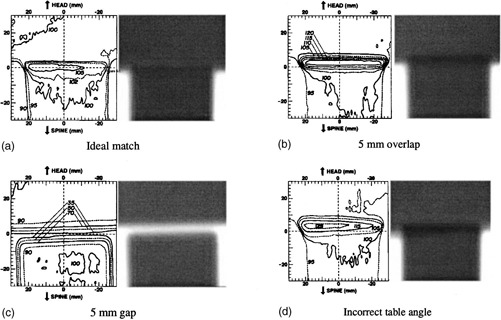
Mini‐verification films have been exposed to “mock” treatments and developed. An optical density scan corresponding to each film and corresponding isodoses for this plane are displayed. (a) An ideal clinical match: a faint thin film of underexposure is present. (b) An intentional overlap causes a dark line corresponding to over exposure. (c) An excessive gap causes a thick light line corresponding to an under‐dosed region. (d) Incorrect table angle causes large irregularly shaped areas of dosimetric inhomogeneity.

(Figures 3a)–3(d) demonstrate the appearance of the mini‐films with corresponding optical density scans. With a clinically acceptable junction, a faint thin line of underexposure is seen at the three‐field junction. The slight dosimetric variations across the junction are related to differences in build up and scatter between the three fields and are not due to any field misplacement or misalignment. Typically there is evidence of handling artifact on the end of films. In the case of a treatment overlap, there is a dark linear region of exposure corresponding to the excessive radiation dose. Because the film is exposed *in situ*, the size of the overlap region correlates closely to the treatment overlap at the patient's neck. Similarly, a linear region of underexposure corresponds to a gap equal to the line's width. If there is an incorrect setup of beam angles, the films can show a “bow‐tie” or wedge‐shaped area of exposure. This would indicate that the cranial fields erroneously diverged into the spine field.

## DISCUSSION

CSI is a complex treatment technique that is difficult to simulate, treat, and verify. Errors in any step of the process could lead to disastrous consequences, such as radiation myelitis. The conventional simulation process with the prone position had been the standard in our department as recently as 1994. It had been our impression that the prone position was uncomfortable for patients and as such, patients would either shift during simulation and/or treatment or even simply be unable to endure the simulation process in a single session. The supine position appears to be much more comfortable for our patients. Although we have not quantified patient satisfaction acceptance of this technique is subjectively better than our older technique. Generally, a 2 to 3 h initial simulation time period was reserved to allow for patient positioning, simulation with filming, fluoroscopy, and documentation.

The CT simulation approach has also reduced patient time for the initial simulation process. Most patients can remain still for the 30–40 min CT scanning session. The actual simulation with field placement and block design takes place on the virtual simulation workstation after the patient has left the department. Mah *et al.* ^4^ has described similar time saving and ease of set up with the use of virtual CT simulation. The CT scans can also be used for three‐dimensional (3D) conformal treatment planning of boost fields. We have used the supine position in nearly all CSI patients, children and adults, since introducing the method in our clinic.

The virtual simulation process is no more complicated than the conventional simulation process. In fact, the flexible large fields‐of‐view and various magnification factors simplifies placement of field boundaries. An entire 40 cm field with margin can be seen on one computer screen. Conventional simulators would require moving the image intensifier during fluoroscopy or taking multiple films to document the entire field length.

The three‐field junction of the cranial ports and posterior spine port can be directly visualized on the virtual simulation monitor. This immediate visual verification helps discover simple arithmetic errors that may prolong the simulation process or worse, allow field placement errors to occur.

Record and verify constraints on the treatment machine are accepted by the treating radiation therapists. Considerable care is given in setting up and aligning patients for daily treatment. Once the patient treatment position has been reproduced, the record and verify computers prevent treatment errors that would result in field overlap or unnecessarily large gaps. We have become so confident with the record and verify constraints during treatment that we now limit the mini‐film verification acquisition to once weekly to correspond to each field junction shift. The independent collimation of the modern linear accelerators allows maintaining the same isocenter during the entire course of CSI. Despite our confidence in our methods of verification, we still employ junction shifts every five fractions in order to prevent error occurring in the same position during an entire treatment course. Patients can still shift slightly during the actual treatment session.

Lastly, the mini‐verification films have been an easy and accurate method of verifying the treatment junction. These films are relatively inexpensive. A sheet of 14×17 in. film will allow junction verification of an entire treatment course once it has been cut to size and placed in the ready pack paper and further enclosed with light occlusive electrical tape. Use of these films predated the installation of our record and verify. Without a record and verify system, we would recommend their use daily. The use of an electronic portal imaging device would complement, not replace, daily mini‐verification films. Additionally, the lead markers at the cervical junction facilitate the clinical review of clinical port films. This gives anatomical confirmation and congruency of the matched fields relative to the cervical spinal cord. Hawkins^5^ has described a similar junction film marker.

In summary, giving up direct visualization of the field junction in the prone position by the light field review is not a disadvantage. The digital couch readings with record and verify, mini‐verification films, and radio‐opaque cervical markers are three tools that overcome this problem.

## CONCLUSION

This integrated approach to simulation, treatment, and verification of CSI is well tolerated by patients. Some of the techniques described here are immediately applicable to other clinics, even those lacking some of the technical features of CT simulation, extended couch treatment windows, record and verify computers, and asymmetric collimation. For example, the lead markers on the immobilization mask at the cervical spine junction can be done in either a prone or supine position. The most critical component of our verification method is the use of the mini‐films. The therapists find them easy to use, and the treating physician and clinical physicist can easily interpret them. These mini‐films allow direct confirmation of the field junction and can be stored as part of the patient's permanent treatment record. These mini‐films can be adapted to verify other multifield junctions techniques.
